# Concentrated growth factors promote epithelization in the mastoid obliteration after canal wall down mastoidectomy

**DOI:** 10.1016/j.bjorl.2025.101561

**Published:** 2025-02-05

**Authors:** Mengyi Liu, Lue Zhang, Quanming Zhang, Nan Zeng, Shuo Li, Shuyue Guo, Yaqin Zhao, Mingxing Tang, Qiong Yang

**Affiliations:** Shenzhen Nanshan People’s Hospital, Department of Otolaryngology, Shenzhen, China

**Keywords:** Cholesteatoma, Mastoid reconstruction, Concentrated growth factor, Mastoidectomy, Mastoid obliteration

## Abstract

**Objective:**

This retrospective study aimed at comparing the extent of epithelialization in mastoid obliteration after Canal Wall Down (CWD) mastoidectomy using Hydroxyapatite (HA) alone or using HA in combination with Concentrated Growth Factor (CGF) extracted from autologous blood.

**Methods:**

A total of 56 patients undergoing the CWD mastoidectomy were enrolled. One group was treated by HA, while the other group received HA and CGF for mastoid obliteration (CGF/HA). A review of the medical follow-up records of all patients was conducted, with a primary focus on the otoendoscopic imaging materials. Complete epithelialization was determined by the absence of granulation, discharge, and swelling. The requisite period for complete epithelialization of the mastoid cavity was thus calculated, scored, and analyzed between treatments.

**Results:**

Ear discharge, graft swelling, and poor blood supply were more frequently observed in the HA group. The proportion of patients group that achieved complete epithelialization within 60 days post operation was significantly higher in the CGF/HA group than the HA group. According to the scoring system for complete epithelialization, the median score of the CGF/HA group was 2, significantly higher than that of the HA treatment group (median score = 1, *p* = 0.032). Altogether, our results indicated that duration needed for complete epithelialization in the CGF/HA group was shorter than the control group.

**Conclusion:**

The application of CGF to surgical cavity obliteration following a CWD mastoidectomy significantly promotes epithelialization, thereby achieving satisfactory results. Randomized controlled trials with a larger number of patients enrolled were worth launching in the future to better substantiate the value of CGF in obliteration of mastoid cavity.

**Level of evidence:**

Level 2-Oxford Centre for Evidence-Based Medicine 2011 Levels of Evidence.

## Highlights

• Large mastoid cavity after CWD mastoidectomy should be obliterated properly.
• HA particles used for cavity obliteration should be properly covered.
• CGF promotes complete epithelialization of the mastoid cavity.

## Introduction

Middle ear cholesteatoma is a common disease present in the department of otorhinolaryngology, with repeated pus and hearing loss in the affected ear as the major clinical manifestations. The pathological feature is characterized by the excessive accumulation of keratinized squamous epithelium in the middle ear or mastoid cavity, causing a local inflammation and progressive injury of the middle ear bone and cause the interruption of the ossicular bone chain. In some severe cases, intracranial and external complications were even observed.^1^ Surgery is recognized as the effective therapy for this disease. Great progress has been made in the operation of cholesteatoma under otoendoscope.^2^ Mastoidectomy is performed for the removal of middle ear cholesteatoma with its invasion into mastoid. Based on whether the posterior wall of the external auditory canal is preserved, two major surgery strategies, Canal Wall Up (CWU) and Canal Wall Down (CWD) mastoidectomy, are commonly used.^3^ Notably, CWU mastoidectomy are prone to residual and/or recurrence of cholesteatoma,^4^ while CWD can reduce the residual and recurrence risks. However, a large mastoid cavity caused by CWD mastoidectomy would induce multiple issues, including the granulation formation of the postoperative cavity wound prolonged healing time, susceptibility to dizziness and/or infection caused by water exposure to the cavity, the need for long-term care to avoid scab buildup, and inability to wear traditional hearing aids,^5^

Thanks to the rapid development of biomaterials in recent years, these problems can be readily addressed by the mastoid obliteration. Either biological or synthetic materials could be used for mastoid obliteration.^6^ Given that the transcanal otoendoscopy approach for mastoidectomy surgery limited the availability of autogenous materials (such as cartilage and pedicled periosteal flap) for mastoid cavity obliteration, artificial materials such as Hydroxyapatite (HA),^7^, ^8^, ^9^ bioactive glass S53P4^10^ and their derivatives^11^, ^12^ have been more commonly used in the CWD mastoidectomy surgery. However, without proper coverage, particles of these synthetic materials would be easy to fall off the mastoid cavity and the risk of infection may still exist.^13^, ^14^

Different from Platelet-Rich Plasma (PRP) and Platelet-Rich Fibrin (PRF), Concentrated Growth Factor (CGF) is the latest generation of blood-derived platelet concentrate, containing various types of growth factors,^15^ which can better promote cell proliferation, migration, and differentiation, as well as angiogenesis and osteogenesis. So far, CGF has been extensively applied in oral, maxillofacial, and nasal surgeries, such as the treatment for the maxillary defects,^16^ implant surgery,^17^ tooth extraction,^18^ nasal septal mucosal defect,^19^ and maxillary sinus lifting.^20^ Favorable postoperative healing effects were observed in these works. Therefore, in this study we employed CGF extracted from the patient's blood as a membrane to cover Hydroxyapatite (HA) to obliterate the cavity following the radical transcanal open mastoidectomy. We found that HA combined with CGF acted as a good substitute of autologous tissue and significantly shortened the time duration required for a complete epithelialization compared the treatment by HA alone.

## Methods

The study was conducted in accordance with the Declaration of Helsinki, and approved by the ethics committee in our hospital with the Institutional Review Board (IRB) number ky-2023-081201. This work is a retrospective analysis that included 56 cases (ears) who underwent CWD mastoidectomy from December 2017 to October 2023. Informed consent forms were obtained from all the involved patients.

### Patient inclusion and exclusion criteria

Adult patients undergoing primary open CWD mastoidectomy for middle ear cholesteatoma were included in this study. Patients admitted from December 2019 to July 2021 received HA to obliterate the mastoid cavity, while GCF began to be used from July 2021 onwards due to its beneficial effect on wound healing following oral, nasal and maxillofacial surgery. The medical follow-up records of all patients up to 6 months were reviewed. While patients that required revision surgery or those that were diagnosed with other preoperative diseases including asthma, diabetes, coagulopathy, cardiovascular disease, basal metabolic disorders, tumors invading into mastoid, chronic liver or kidney disease and systematic infections were excluded.

### Pre-operative examination

**Fig. 1:**
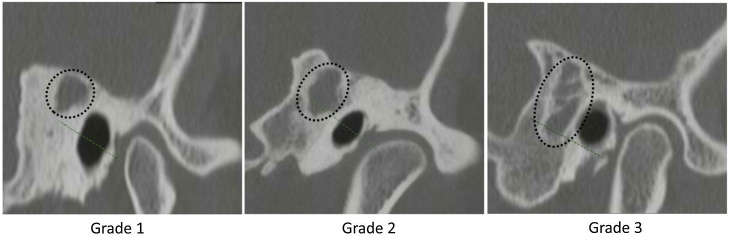
The Computed Tomography (CT) images of temporal bone in the coronal plane revealed the position and size of cholesteatoma marked by the dotted line circles, which were scored as Grade 1, 2 and 3 in this study.

Preoperative otoendoscopic examination was firstly performed, revealing the retraction pocket in the pars tensa together with accumulation of white or brown keratin debris in the pars flaccida or at its junction area with the pars tensa. Audiometric threshold of pure tone was measured to determine the chi bone space. Computed Tomography scan of the temporal bone was performed to determine the cholesteatoma position and to record its size. The inferior border of the cholesteatoma is used as a demarcation line, whereby those that do not exceed the midline of the external auditory canal are classified as grade 1, those that exceed the midline of the external auditory canal but do not extend beyond the lower wall of the external auditory canal are classified as grade 2, and those that extend beyond the lower wall of the external auditory canal to the tip of the mastoid are classified as grade 3 (Fig. 1).

### CGF preparation

**Fig. 2:**
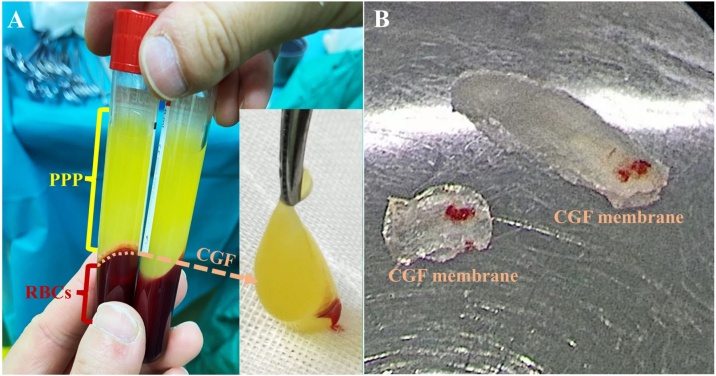
(A) Variable speed centrifugation of patient’s blood samples was used to separate the section containing Concentrated Growth Factor (CGF) from Platelet Poor Plasma (PPP) and Red Blood Cells (RBCs). (B) The CGF membrane was formed by squeezing with sterilized, skimmed gauze.

As previously described,^21^ 9 mL venous blood from each patient was collected and stored in sterile vacuum tube (Greiner Bio-One, GmbH, Kremsmunster, Austria) without any anticoagulant. Then, the tube was immediately centrifuged (Medifuge, Silfradentsrl, Italy) with a fixed process: acceleration for 30 seconds (s), 2700 rounds per minutes (rpm) for 2 minutes (min), 2400 rpm for 4 min, 2700 rpm for 4 min, 3000 rpm for 3 min, deceleration to a stop for 36 seconds. Three sections were thus generated, including an upper layer consisting of Platelet Poor Plasma (PPP), a middle layer of light yellow gelatin containing CGF, and a lower layer containing the Red Blood Cells (RBCs) (Fig. 2A). CGF was obtained with sterile tweezers from each tube. The lower RBCs were cut away using the surgical tissue scissors (319754, SHINVA, China), and the CGF membranes (Fig. 2B) were formed by manually squeezing using the sterilized, skimmed gauze (with 50 × 70 mm in size).^22^

### Procedures for mastoidectomy and mastoid obliteration

**Fig. 3:**
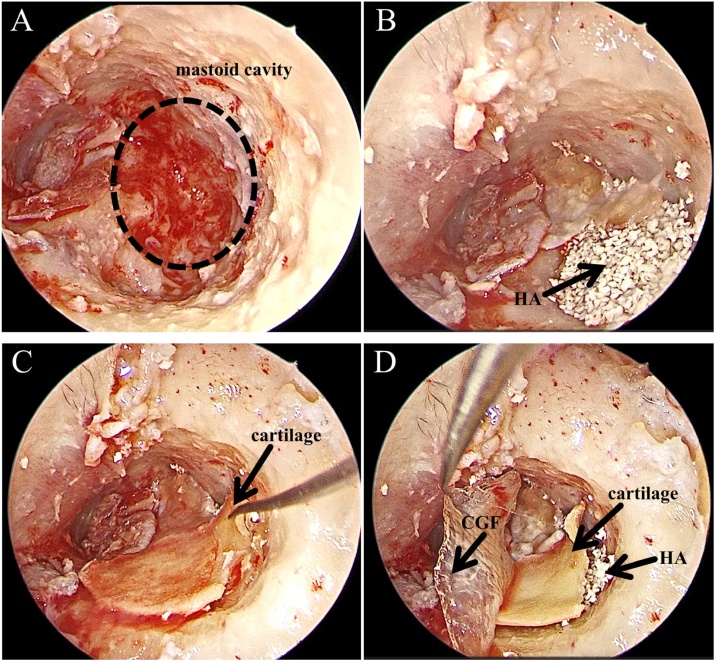
(A) Cavity formed by CWD mastoidectomy (circled). (B) Cavity filled with HA (arrow point). (C) The mastoid cavity filled with HA was covered by the tragus cartilage. (D) The CGF membrane was added as the cover on HA and the tragus cartilage.

Surgeries for all the patients were mainly performed by one surgeon, who was the chief director in the Department of Otorhinolaryngology with over 25 years of surgery experience. Firstly, local injection of lidocaine epinephrine saline around the ear and inside the ear canal. Tragus cartilage was excised to extract the perichondrium for tympanoplasty. Endomeatal incisions were made to lift the tympanomeatal flap and the fibrous anulus, allowing the surgeon to have a direct vision of the middle tympanic chamber. The external ear canal was firstly enlarged by continuous perfusion mode under otoendoscopy. The endoscopic underwater bone drilling technique was then adopted to remove the bone along the edge of the cholesteatoma, and open the upper tympanic cavity, tympanic sinus, and mastoid (Fig. 3A). Tympanum, ossicular chain and tympanic orifice were examined. The tragus-perichondrium was taken to reconstruct the tympanic air space. For the HA group, the mastoid cavity was firstly filled with HA (Fig. 3B), followed by coverage with the tragus cartilage, and the tympanomeatal flap was reset (Fig. 3C). For the CGF/HA group, the procedure was the same as in the HA group but with the use of CGF as a cover membrane on HA particles (Fig. 3D). Finally, the tragus incision was sutured, and the EAC was filled with antibiotic-loaded gelatine sponges to ensure close attachment of the skin flap to the bone surface. The EAC opening was filled with antibiotic gauze, and the cavum conchae was filled with a cotton ball. Ears of all patients were checked at day 20 post operation for the first return visit and every 2 weeks after that until the intraoperative cavity was completely epithelialized.

### Evaluation of complete epithelialization

Complete epithelialization was defined as an entire epithelial coverage of the mastoid cavity at the incision site with normal blood supply while granulation, dampness, swelling, infection, and HA shedding were not observed. A published scoring criteria for evaluating epithelialization of postoperative cavity with moderate modifications was used in this study.^23^ Complete epithelialization time was achieved within 30 days would be scored as 3, while the duration ranging from 30 to 60 days, 60 to 90 days and more than 90 days would be scored as 2, 1, and 0, respectively.

### Data statistics

The data was analyzed using SPSS Statistics 22.0 (IBM, USA). The Chi-square test was used to compare categorical variables between groups. The normality of the quantitative variables was assessed by the Shapiro–Wilk test. Non-normal variables between groups were analyzed using the Mann–Whitney *U* test; *p-*value less than 0.05 was considered significant.

## Results

### General information

In this study, information regarding the gender and age of patients, as well as the lateral side and size of cholesteatoma from two treatment groups were recorded and compared. There were 14 men (51.9%) and 13 women (48.1%) in the CGF/HA group, while 21 men (72.4%) and 8 women (27.6%) in the HA group (Table 1). No significant difference in gender was found between groups (χ^2^ = 2.522, *p* = 0.112). In addition, no significant lateral difference was observed, as the CGF/HA group involved 13 left ears (48.15%) and 14 right ears (51.85%) while the HA group included 11 left ears (37.93%) and 18 right ears (62.07%; χ^2^ = 0. 596, *p* = 0.440). The size of cholesteatoma between two groups did not differ (χ^2^ = 1.107, *p* = 0.576), either (Table 1). The median age of the CGF/HA group was 40.0 (27.0, 51.0), which was not significantly different (*p* = 0.628) from that of HA group (Table 1).

**Table 1:** Patient demographics and characteristics by treatment group. Chi-Square or Mann Whitney *U* test was used to analyze the data.

Characteristics		Group		χ^2^ / Mann–Whitney value	*p-*value
		HA	CGF/HA		
Gender	Man	21 (72.40%)^a^	14 (51.90%)^a^	2.522	0.112
	Women	8 (27.60%)^a^	13 (48.10%)^a^		
Age		36.0 (27.8, 45.3)^b^	40.0 (27.0, 51.0)^b^	380.5	0.628
Lateral side	Left	11 (37.93%)^a^	13 (48.15%)^a^	0.596	0.440
	Right	18 (62.07%)^a^	14 (51.85%)^a^		
Cholesteatoma size	Grade 1	5 (17.24%)^a^	4 (14.81%)^a^	1.104	0.576
	Grade 2	17 (58.62%)^a^	13 (48.15%) a		
	Grade 3	7 (24.14%)^a^	10 (37.04%)^a^		
Postoperative ear discharge		2 (6.90%)^a^	0 (0%)^a^	0.448	0.503
Postoperative HA shedding		1 (3.45%)^a^	0 (0%)^a^	0.48	0.518

HA, Hydroxyapatite; CGF, Concentrated Growth Factor.

a Data were presented as the frequency number with their proportions (shown in brackets) within the whole group. b, data were presented as the median, with the 25% and 75% percentile numbers shown in brackets.

### Otoendoscopic examination suggested the promoting effect of CGF on complete epithelization

The recovery status recorded using otoendoscopy in all the follow-up visits, starting from 20 days post operation until the completion of epithelialization, was reviewed. During all the follow-up process that lasted 7 months minimally to 6 years maximumly, we did not find any recurrence or residual of cholesteatoma in both groups. Moreover, other ear complications such as vertigo, tinnitus or hearing loss were not found.

**Fig. 4:**
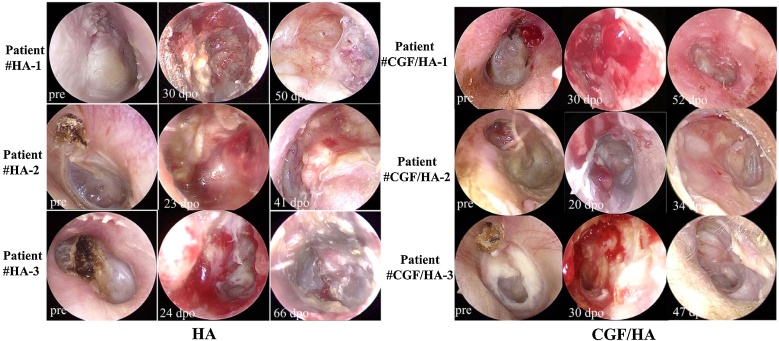
Preoperative and postoperative endoscopic examination of 3 presentative cases in the HA group and CGF/HA group. Pre means pre-operation, dpo means days post operation.

In most ears from the HA group at the first follow-up visit, granulation was frequently found in incision sites and the central area of the mastoid operative cavity graft (Fig. 4). The epithelium appeared pale with swelling graft. Ear discharge causing a moist operative cavity was found in two cases (Table 1). Only one case had partially HA particle shedding, but a revision surgery is not required (Table 1). In the CGF/HA group, however, granulation tissue was found at the incision site, but not in the mastoid operative cavity. The operative cavity was completely covered by epithelial tissue with normal blood supply (Fig. 4).

### Statistical analysis confirming the efficacy of CGF in promoting the epithelialization

**Fig. 5:**
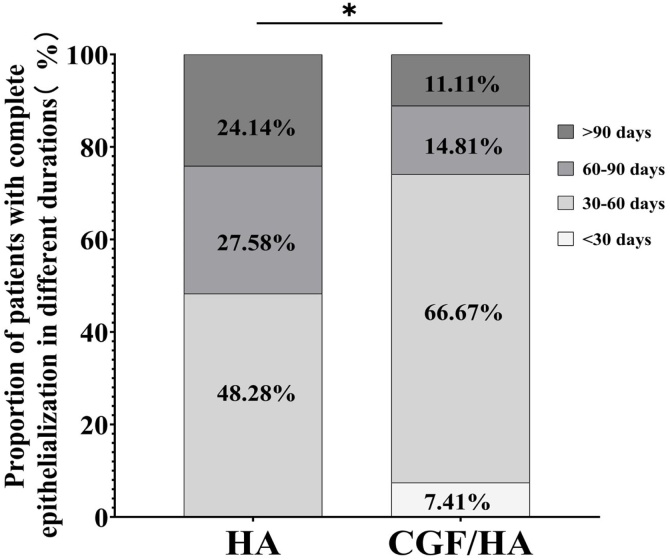
The proportion of patients with different time for postoperative epithelialization in the HA treated group and the group treated by HA combined with CGF (*The *p-*value less than 0.05).

The above otoendoscopic observation results suggested that CGF may significantly promote the completion of the epithelialization of the operative cavity after radical mastoidectomy. To substantiate this, we collected all the otoendoscopic image data from the follow-up visits and conducted a systematic statistical comparison of their recovery status between two groups. To achieve a complete epithelialization in the CGF/HA group, 7.4% of the patients (2/27) needed less than 30 days after operation, 66.67% of the patients (20/27) required 30–60 days, 14.81% for 60–90 days, and 11.11% for more than 90 days. By contrast, none in the HA group had a complete epithelialization within 30 days, while 48.3% of the patients (14/29) required a period of 30–60 days, 27.58% for 60–90 days, and 24.14% for more than 90 days (Fig. 4). Though Chi-Square test, we found that the proportion of patients achieving complete epithelialization within 60 days in the HA/CGF group was significantly higher than that of the HA group (χ^2^ = 3.901, *p* = 0.048) (Fig. 5).

**Fig. 6:**
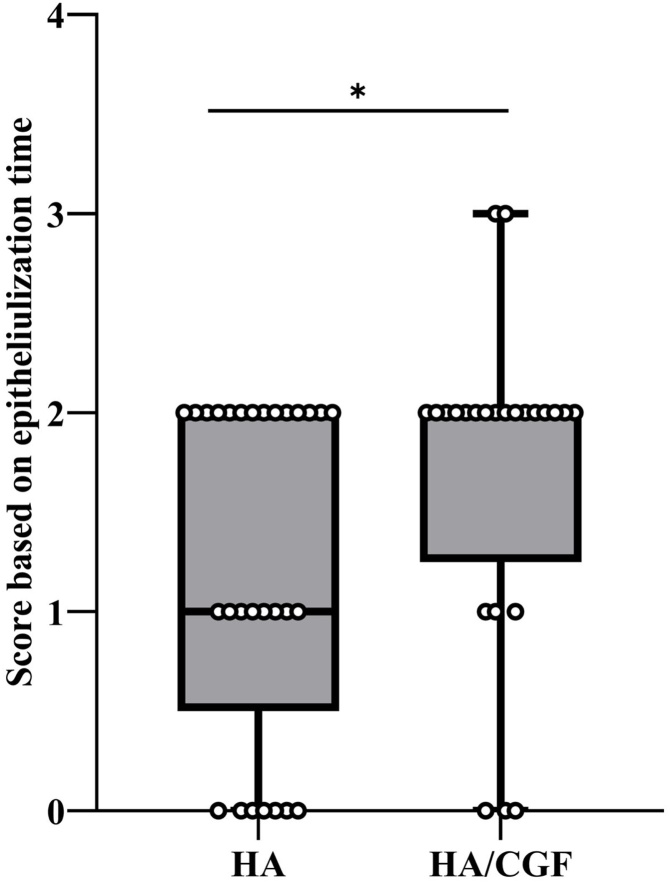
Epithelialization scores of the HA group and the CGF/HA group (*Indicates significant difference in epithelialization scores between the two groups with the *p-*value less than 0.05).

To better assess the recovery status, we adopted a previously published scoring method but with moderate modification to evaluate the duration required for complete epithelialization.^23^ The overall difference between two treatment groups was therefore determined. We found that the median score of the CGF/HA group was significantly lower than that of the HA group (*p* = 0.032) (Fig. 6), reinforcing the effect of CGF in promoting the epithelization.

## Discussion

In this work, a total of 56 patients were enrolled and divided into two groups, depending on whether CGF is used for obliteration of the mastoid cavity. Through Chi-Square test, we found no difference in gender and age of patients as well as lateral side and size of cholesteatoma between groups, suggesting that no bias across groups exists. This laid a solid foundation for an unbiased evaluation of the CGF efficacy on epithelization.

Here we employed the autogenous blood derived CGFs together with HA for mastoid obliteration. The artificial materials, such as bioactive glass, HA and their derivatives have been widely used for mastoid obliteration after CWD mastoidectomy.^7^, ^9^, ^11^, ^12^ However, if not properly covered, it would be easy to fall off the mastoid cavity, and its exposure to air may increase the risk of infection. The commonest strategy to optimize the outcome of cavity obliteration is to employ the fascia, perichondrium, or cartilage as a film to cover the exposed HA and mastoid cavity. However, the transcanal approach for the mastoidectomy surgery limited the availability of such autogenous soft tissues. In the field of dental implantology, HA and CGF are employed to fill bone defects in alveolar bone, thereby providing a robust foundation for secondary root fixation.^24^, ^25^ As the third generation of products derived from blood, CGF can be prepared with relative ease using variable speed centrifugation technology, which has rendered it an optimal option for mastoid reconstruction.

Although CGF has been widely used in stomatology, dental implantology, maxillofacial surgery bone defect and regeneration of nasal epithelium,^18^, ^19^, ^24^, ^26^^,^^27^ our work is the first study that reports the application of CGF in promoting the mastoid cavity epithelization in the field of otorhinolaryngology. Our initial otoendoscopic observation showed that within 60 days post operation many ears treated by HA were characterized by wet ear discharge, pale ear canal epithelium, and graft swelling, indicative of poor blood supply and a higher risk of infection, while these traits were less frequently found in the CGF/HA group. Subsequent statistical analysis substantiated the promoting effect of CGF on the complete epithelization of mastoid cavity.

The major basis of CGFs to promote epithelization is that the CGF film provides a three-dimensional space for adhesion and proliferation of white blood cells and platelets^28^, ^29^, ^30^ and a prolonged release of various growth factors.^15^ The CGF membrane is composed of several growth factors contributing to vascular maintenance^31^ and angiogenesis,^32^, ^33^ including Platelet-Derived Growth Factor (PDGF), Insulin-Like Growth Factors (IGF-1), transforming growth factor-β1 (TGF-β1), and Vascular Endothelial Growth Factor (VEGF). CGFs also contain Epidermal Growth Factor (EGF), which is able to regulate the proliferation and migration of epithelial cells and promote the growth of various epidermal tissues.^34^ Additionally, the three-dimensional reticular fibrin in CGF is rich in Interleukins (IL-1, IL-4, IL-6) and CD34 + immune cells,^22^ which can effectively prevent infection^35^ and sustain the immune homeostasis. The close contact of CGF with the incision site would facilitate the release of those growth factors, thus explaining why CGFs can rapidly promote epithelium growth, restore blood supply, and reduce the risk of infection.

Another advantage of CGF is that it is derived from the patient's own venous blood, which dispel the concern about tissue rejection. As soft tissue sampling is limited through the natural orifices of the ear canal in endoscopic ear surgery, the tragus-perichondrium needs to be used for tympanoplasty reconstruction of the tympanoplasty. The auxiliary coverage of HA by CGF membrane therefore reduces the necessity of obtaining temporal muscle fascia through retroauricular incision, saves operative time, and meets the cosmetic needs of patients.

It should be noted that limitations of this study still exist. The biggest one is the small scale of enrolled patients. Despite of extensive efforts, only a total of 56 patients were recruited because of a stringent inclusion criterion we set in this retrospective work. A large number of cases undergone revision surgery and those with incomplete or unstandardized follow-up history had been excluded. Another limitation of this work is the difficulty of accuracy in determining the duration of complete epithelization, as most patients were followed up every two weeks, or longer. Reducing the time interval between each return visit could help to provide a more detailed dynamic information for the epithelization of mastoid cavity, but it will also cause a burden in our labor work.

## Conclusion

Based on the findings from the present study, application of CGF in mastoid cavity obliteration can effectively promote tissue healing, shorten the time of postoperative mastoid cavity epithelialization, reduce the financial burden of patients, and improve their quality of life. Therefore, CGF could be an ideal material for mastoid cavity obliteration. However, larger scale randomized controlled trials are still needed in the future to better validate the beneficial values of CGF. Cases with underlying or chronic co-morbidities such as metabolic diseases or infections should be carefully considered or excluded, as they may interfere with the evaluation of CGF effect.

## CRediT authorship contribution statement

All the authors contributed to this work. Material preparation, data collection and analysis were performed by Mengyi Liu, Mingxing Tang, Qiong Yang. The manuscript was written by Mengyi Liu, Mingxing Tang and Qiong Yang.

## Ethical

The study was conducted in accordance with the Declaration of Helsinki, and

approved by the Department of Otolaryngology, Huazhong University of Science and

Technology Union Shenzhen Hospital (ethical code ky-2023-081201).

## Funding

This work was supported by the Shenzhen Science and Technology Innovation Commission for Research and Development Projects (JCYJ20220530141616037 andJCYJ20230807115827057), the Outstanding Young Researcher program (NSZD2023011) and the key program (NS2022005) granted by the Science and Technology Key Research Program in Nanshan District Health Care System and the Medical Research Foundation of Guangdong Province (A2022046).

## Declaration of competing interest

The authors declare no conflicts of interest.
